# Cobalt(II)-Mediated Fenton-like Reactions: Effects of Second-Sphere H_2_O_2_ and Thiolate Coordination

**DOI:** 10.1021/acs.inorgchem.5c04687

**Published:** 2025-12-16

**Authors:** Hsing-Yin Chen, Yu-Fen Lin

**Affiliations:** Department of Medicinal and Applied Chemistry, 38023 Kaohsiung Medical University, Kaohsiung 80708, Taiwan

## Abstract

While the Co­(II) aqua complex is not a good catalyst for H_2_O_2_ decomposition due to its high redox potential, the Fenton-like activity of Co­(II) can be promoted by chelation with suitable ligands. Previous experiments have shown that different reactive oxygen species (ROS) are generated in the presence of different ligands, but the underlying mechanism is unclear. In this study, density functional theory calculations are used to investigate the decomposition of H_2_O_2_ mediated by Co­(II) complexes containing nitrilotriacetate (NTA), ethylenediaminetetraacetate (EDTA), and glutathione (GSH). For the NTA– and EDTA–Co­(II) complexes, the formation of free ^•^OH via the *conventional* Fenton-like pathway is thermodynamically unfavorable. However, H_2_O_2_ accumulated in the second coordination sphere via hydrogen bonding with carboxylate groups can readily undergo hydrogen atom transfer with ^•^OH produced from the coordinated H_2_O_2_, generating ^•^OOH as the major ROS. This reaction step provides a thermodynamic driving force for the H_2_O_2_ decomposition, which we call the *second-sphere H*_2_ *O*_2_ *-assisted* Fenton-like reaction. On the other hand, the *conventional* Fenton-like reaction of the GSH–Co­(II) complex is kinetically and thermodynamically favorable, generating ^•^OH as the major ROS. Detailed analysis reveals that the thiolate group of GSH plays a dominant role in promoting the *conventional* Fenton-like reaction.

## Graphical abstract

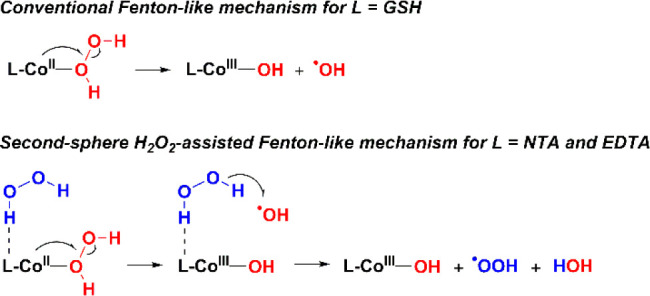

## 1 Introduction

Transition metal ion-mediated hydrogen peroxide (H_2_O_2_) activation, known as Fenton (Fe^2+^ aqua complex) and Fenton-like (other metal complexes) reactions, plays a role in biological oxidation processes and is thought to be associated with aging and human diseases such as neurodegenerative diseases,^1,2^ cardiovascular diseases,^3,4^ and cancers.^5,6^ On the other hand, utilizing the weak acidity and H_2_O_2_ overproduction of the tumor microenvironment, Fenton and Fenton-like reactions have been exploited to develop an emerging cancer treatment strategy called chemodynamic therapy.^7–9^ Also, the strong oxidizing power of Fenton and Fenton-like reactions can be directly applied to wastewater treatment.^10,11^

Cobalt is a vital micronutrient for human beings. It is a key component of vitamin B_12_ (i.e., cobalamin), which is essential to metabolism. On the other hand, elevated cobalt concentrations in the blood have been reported to have adverse health effects.^12,13^ Co^2+^_aq_ possesses a standard electrode potential [*E*°(Co^3+^/Co^2+^) = 1.92 V] much higher than that of H_2_O_2_ [*E*°(H_2_O_2_,H^+^/^•^OH,H_2_O) = 0.71 V] and, therefore, cannot reduce H_2_O_2_ to proceed with the conventional Fenton-like (Reaction 1).

1 Coaq2++H2O2→Coaq3++OH•+OH−

Experimental studies have shown that the efficacy of Co^2+^_aq_ in activating H_2_O_2_ is lower than that of Fe^2+^_aq_ and the reactive oxygen species (ROS) produced by the two systems are different,^14–24^ suggesting that the activation mechanisms in the two systems are different. A recent density functional theory (DFT) study revealed that the Co^2+^_aq_-mediated H_2_O_2_ activation was initiated by reacting with two H_2_O_2_ molecules to form the [(H_2_O)_4_Co^II^(OOH)­(H_2_O_2_)]^+^ complex.^25^ Depending on the relative orientation between the ^–^OOH and H_2_O_2_ ligands, this complex decomposes via different pathways, leading to the formation of ^•^OOH/O_2_^•–^, [(H_2_O)_5_Co^III^(O)]^+^ (i.e., crypto-^•^OH), ^3^O_2_, and HOOOH or Co^II^–OOOH intermediates. The latter two intermediates are rapidly hydrolyzed to singlet oxygen ^1^O_2_.^25^ Considering the low H_2_O_2_ concentration in vivo, the possibility of Co^2+^_aq_ reacting with two H_2_O_2_ molecules is small, so the role of Co^2+^_aq_-mediated Fenton-like reactions in cobalt toxicity should be negligible.

However, it is known that ligand complexation may alter the redox potential and thus the reactivity of the metal ions. For instance, Moorhouse et al. demonstrated that the addition of the chelating agent ethylenediaminetetraacetate (EDTA) enhanced the hydroxylation of aromatic compounds and the degradation of deoxyribose by the Co­(II)/H_2_O_2_ reaction. Scavenger experiments further indicated that in the presence of EDTA, free ^•^OH was formed, whereas in the absence of EDTA, ^•^OH was formed in a site-specific manner (i.e., crypto-^•^OH).^15^ The formation of free ^•^OH in the Co­(II)/H_2_O_2_/EDTA system was later confirmed by Eberhardt et al., using dimethyl sulfoxide (DMSO) as a probe. The study also confirmed the formation of Co­(III) during the reaction by spectroscopy.^26^ The formation of free ^•^OH and Co­(III) suggests that the EDTA–Co­(II) complex reacts with H_2_O_2_ in a Fenton-like manner (eq 1). However, only a small amount of the consumed H_2_O_2_ was converted to ^•^OH; the authors inferred that there was another dominant decomposition pathway for H_2_O_2_.^26^ Hanna et al. used the electron paramagnetic resonance (EPR) spin trapping technique with 5,5-dimethyl-1-pyrroline-*N*-oxide (DMPO) to detect the ROS formation from the reactions of Co­(II) with H_2_O_2_ in the absence and presence of nitrilotriacetate (NTA). It was found that the amount of DMPO–^•^OOH adduct produced in the Co­(II)/H_2_O_2_/NTA system was significantly higher than that in the Co­(II)/H_2_O_2_ system. In the presence of NTA, DMPO–^•^OH adduct was also detected, and the appearance of Co­(III) was confirmed by spectroscopy.^21^ EPR spin trapping experiments have also been used to investigate the Co­(II)/H_2_O_2_/glutathione (GSH) system. Shi et al. discovered that the addition of GSH enhanced the ^•^OH generation from the reaction of Co­(II) with H_2_O_2_. The formation of glutathionyl radical (GS^•^) was also detected, which was attributed to the site-specific reaction between GSH and ^•^OH produced from H_2_O_2_ by the GSH–Co­(II) complex.^27^ Recently, Li et al. published a series of papers describing the use of tris­(hydroxymethyl)­aminomethane (Tris) to promote persistent generation of ^•^OH from Co­(II)/H_2_O_2_ reactions.^22–24^ They demonstrated that the Co^2+^/H_2_O_2_/Tris system can be applied in pollutant degradation,^22^ bacterial inactivation,^23^ and long-lasting chemiluminescence.^24^

Due to historical reasons, the iron-based Fenton reaction has been extensively studied since its discovery in 1894. However, the behavior of the Fenton reaction is influenced by a variety of factors, such as reagent concentration, pH conditions, and the properties of ligands and buffers. Therefore, its detailed mechanism has only gradually become clear in the last two decades. For example, whether the ROS generated by the Fenton reaction is ^•^OH or Fe­(IV) species has been a long-standing issue of contention.^28–34^ This debate was not settled until 2003 to 2012, during which time a series of experiments revealed the pH dependence of ROS generation in the Fenton reaction,^35–38^ and the theoretical basis for this pH-dependent behavior did not emerge until 2018.^39–41^ To this day, the detailed mechanism of the Fenton reaction is still under investigation.^42^

In recent years, the application of cobalt-based Fenton-like systems has been significantly increasing,^14,19,22,23,43–45^ yet our mechanistic understanding of the cobalt-based Fenton-like reactions lags far behind that of the iron-based Fenton-like reactions. As mentioned above, why does NTA promote the formation of ^•^OOH, whereas GSH promotes the formation of ^•^OH in the Co­(II)/H_2_O_2_ reaction? And why is it that only 10–15% of the consumed H_2_O_2_ is converted into ^•^OH in the EDTA/Co­(II)/H_2_O_2_ system? Does this system have another major H_2_O_2_ decomposition pathway? If so, what is this pathway and what is the ROS produced? These questions remain open. Understanding how ligands affect Fenton-like reactivity and how they lead to selective production of specific ROS is crucial for expanding the applications of Fenton-like systems. Therefore, we decided to conduct a DFT study to clarify the above issues.

## 2 Computational Methods

The hybrid τ-dependent gradient-corrected functional TPSSh^46,47^ in combination with triple-ζ quality basis sets Def2-TZVP^48,49^ was used for the present DFT calculations. Geometry optimizations and vibrational frequency calculations were carried out in an aqueous environment treated with the SMD solvation model.^50^ The SMD-TPSSh/Def2-TZVP method was selected because it has been demonstrated to provide the most satisfactory result for the standard electrode potential of Co­(III)/Co­(II) in our previous benchmark study.^25^ Intrinsic reaction coordinate (IRC) calculations were performed on the resulting transition states to confirm that they were correctly connected to the desired intermediates. The Wertz correction was used to amend the error in solution entropy caused by using gas-phase statistical thermodynamics formulas in standard quantum chemical calculations.^25,51–54^ According to this approach, the absolute entropies of 1 M solutes and 55.5 M H_2_O in aqueous solutions were calculated by eqs 2 and 3, respectively.

2 $$S_{s}=0.54S_{\mathrm{gas}}^{{}^{\circ}}+\left(0.24\mathrm{cal}\cdot {\mathrm{mol}}^{-1}\cdot K^{-1}\right)$$

3 $$S_{s}=0.54S_{\mathrm{gas}}^{{}^{\circ}}-\left(7.74\mathrm{cal}\cdot {\mathrm{mol}}^{-1}\cdot K^{-1}\right)$$

All calculations were accomplished by using the Gaussian 16 progam.^55^

Since the ligands investigated are biologically relevant and the related experimental studies were conducted under physiological conditions at pH 7.2–7.4, the model structures used in this study were selected to correspond to neutral pH conditions.

## 3 Results and Discussion

### 3.1 Activation of H_2_O_2_ by NTA–Co­(II) Complex

Figure 1 presents the computational results of the conventional Fenton-like reaction mediated by the NTA complex of Co­(II). Calculations show that the tetradentate ligand NTA chelates with Co­(II) to form a five-coordinate trigonal bipyramidal [(NTA)­Co^II^(H_2_O)]^−^ complex (**SC**_**N**_ **1**), in which an exchangeable water ligand occupies the axial position. We attempted to optimize the six-coordinate octahedral [(NTA)­Co^II^(H_2_O)_2_]^−^ complex but failed; during the geometry optimization, one of the H_2_O ligands spontaneously moved into the second coordination sphere, restoring the five-coordinate [(NTA)­Co^II^(H_2_O)]^−^·H_2_O structure. Both octahedral and trigonal bipyramidal structures of NTA complexes of Co­(II) have been reported. Battaglia et al. and Zasurskaya et al. reported an octahedral structure in which Co­(II) is coordinated by the nitrogen and three carboxylate oxygens from one NTA ligand, one carboxylate oxygen from a neighboring NTA ligand, and one water molecule.^56,57^ Another octahedral structure reported by Zhang et al. involves a nitrogen and three carboxylate oxygens from one NTA ligand and two carboxylate oxygens from two adjacent NTA ligands.^58^ It is noteworthy that all of these six-coordinate octahedral structures involve sharing carboxylate groups with adjacent complexes, which may be due to crystal packing effects. On the other hand, Polyakova et al. synthesized three [(NTA)­Co^II^X]^2–^ complexes (X = Cl^–^, Br^–^, and NCS^–^). These complexes have similar trigonal bipyramidal structures, in which Co­(II) is surrounded by a nitrogen and three oxygens of one NTA ligand and an X anion in the *trans* position with respect to N.^59^ The fact that NCS^–^ is a stronger ligand compared to H_2_O and [(NTA)­Co^II^(NCS)]^2–^ exhibits a five-coordinate trigonal bipyramidal geometry supports our computational result that six-coordinate octahedral [(NTA)­Co^II^(H_2_O)_2_]^−^ is not a stable structure and five-coordinate trigonal bipyramidal [(NTA)­Co^II^(H_2_O)]^−^ (**SC**_**N**_ **1**) is a reasonable starting complex for the Fenton-like reaction. We also considered the possibility of the side-on coordination of H_2_O_2_ in the NTA–Co­(II) complex. A potential energy surface (PES) scan shows that the energy monotonically increases as H_2_O_2_ approaches the Co­(II) center via a side-on manner, suggesting that there is no stable side-on η^2^-[(NTA)­Co^II^(H_2_O_2_)]^−^ structure (Figure S1).

**Figure 1:**
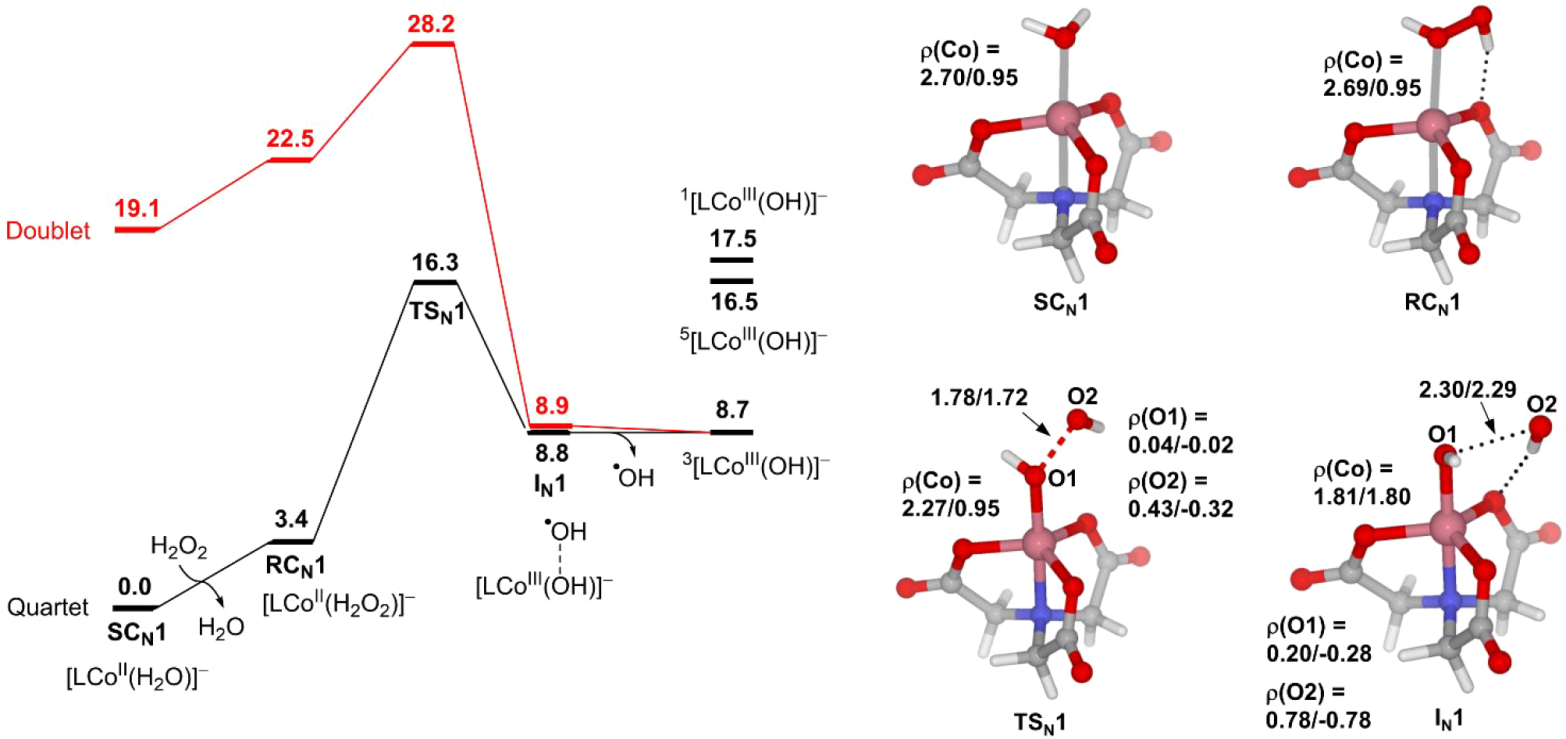
Free energy profile of the conventional Fenton-like reaction mediated by the NTA complex of Co­(II). Selected bond distances and spin densities (ρ) are given for quartet/doublet states. The energy unit is kcal/mol, and the bond distance unit is Å.

Starting from **SC**_**N**_ **1**, the first step is the substitution of H_2_O_2_ for H_2_O, generating the reactant complex [(NTA)­Co^II^(H_2_O_2_)]^−^ (**RC**_**N**_ **1**). The ligand exchange is assumed to take place via the dissociative mechanism.^60,61^ A PES scan indicates that the dissociation of the water ligand in Co­(II) complexes is a rapid process with an energy barrier not exceeding 5.3 kcal/mol (Figures S2–S5). The H_2_O_2_ in **RC**_**N**_ **1** then undergoes reductive O–O bond homolysis to produce the intermediate [(NTA)­Co^III^(OH)]^−^·^•^OH (**I**_**N**_ **1**), which further dissociates into [(NTA)­Co^III^(OH)]^−^ and free ^•^OH. We notice that the high-spin (HS) quartet state and the low-spin (LS) doublet state of **I**_**N**_ **1** are isoenergetic. Spin population analysis indicates that ^**4**^ **I**_**N**_ **1** and ^**2**^ **I**_**N**_ **1** have the same middle-spin (MS) triplet state [(NTA)­Co^III^(OH)]^−^ moiety, but they couple to the leaving ^•^OH in different ways. In ^4^ **I**_**N**_ **1**, the spins on the two fragments are ferromagnetically coupled [ρ­(Co) = 1.81; ρ­(^•^OH) = 0.78], whereas in ^**2**^ **I**_**N**_ **1**, they are antiferromagnetically coupled [ρ­(Co) = 1.80; ρ­(^•^OH) = −0.78]. The similar electronic structures and energies of ^**4**^ **I**_**N**_ **1** and ^**2**^ **I**_**N**_ **1** can be easily understood by looking at the d-orbital splitting diagram of the trigonal bipyramidal complex shown in Figure 2.

**Figure 2:**
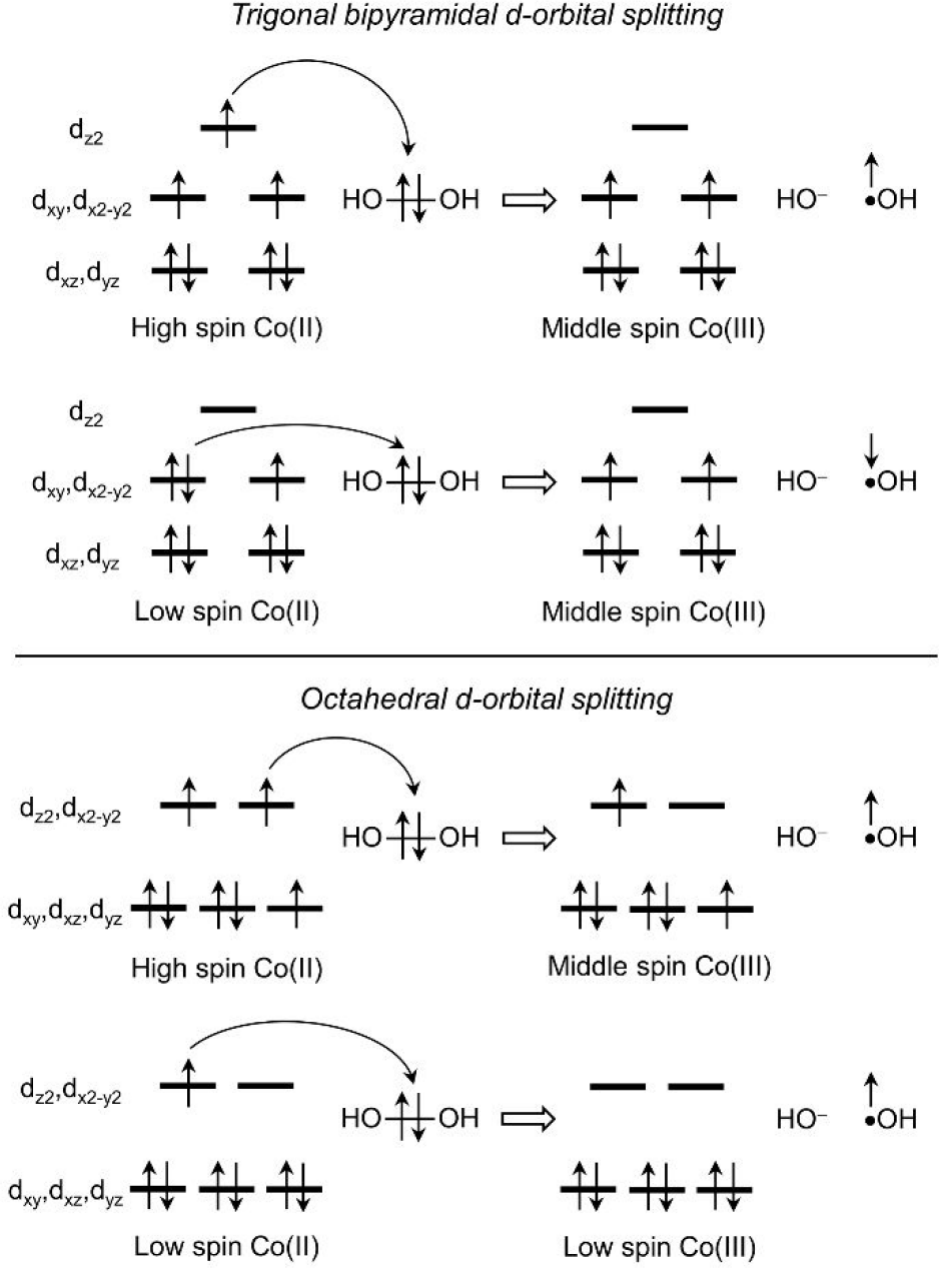
Electronic structure changes during Fenton-like reactions mediated by five-coordinate trigonal bipyramidal and six-coordinate octahedral Co­(II) complexes.

Although the conventional Fenton-like reaction between H_2_O_2_ and [(NTA)­Co^II^(H_2_O)]^−^ is kinetically feasible (Δ*G* ^‡^ = 16.3 kcal/mol), the reaction is endergonic by ∼9 kcal/mol, indicating that this H_2_O_2_ activation pathway is not efficient. In our previous study of the NTA/Fe/H_2_O_2_ system, we found that H_2_O_2_, which is more acidic than H_2_O, inclines to bind with the carboxylic groups of NTA through hydrogen bonding. The H_2_O_2_ accumulated in the second coordination sphere can readily react with the oxo group of Fe^IV^=O.^54^ Inspired by this previous finding, we next considered the reaction of H_2_O_2_ with the model complex [(NTA)­Co^II^(H_2_O)]^−^·H_2_O_2_ (**SC**_**N**_ **2** in Figure 3), which includes a H_2_O_2_ molecule in the second coordination sphere. In **SC**_**N**_ **2**, H_2_O_2_ in the second coordination sphere acts as a proton donor and a proton acceptor to form hydrogen bonds with the carboxylic group and the water ligand, respectively. The water ligand of **SC**_**N**_ **2** is then replaced by another H_2_O_2_ to form the reactant complex [(NTA)­Co^II^(H_2_O_2_)]^−^·H_2_O_2_ (**RC**_**N**_ **2**). The H_2_O_2_ coordinated to Co­(II) subsequently undergoes O–O bond cleavage to generate the intermediate [(NTA)­Co^III^(OH)]^−^·^•^OH·H_2_O_2_ (**I**_**N**_ **2**). So far, the activation process and the corresponding energies are very close to the results without H_2_O_2_ in the second coordination sphere (cf. Figures 1 and 3). However, calculations indicate that leaving ^•^OH in **I**_**N**_ **2** will be rapidly reduced by the second-sphere H_2_O_2_ via hydrogen atom transfer (HAT), producing the intermediate [(NTA)­Co^III^(OH)]^−^·H_2_O·HOO^•^ (**I**_**N**_ **3**). This reaction step provides the thermodynamic driving force, making the overall reaction highly exergonic, and thus prevents the recombination of ^•^OH with Co^III^–OH (i.e., the reverse reaction **I**_**N**_ **2** → **RC**_**N**_ **2**). This second-sphere H_2_O_2_-assisted Fenton-like pathway accounts for the experimental observation that the amount of DMPO–^•^OOH adduct produced in the Co­(II)/H_2_O_2_/NTA system is significantly higher than that in the Co­(II)/H_2_O_2_ system.^21^

**Figure 3:**
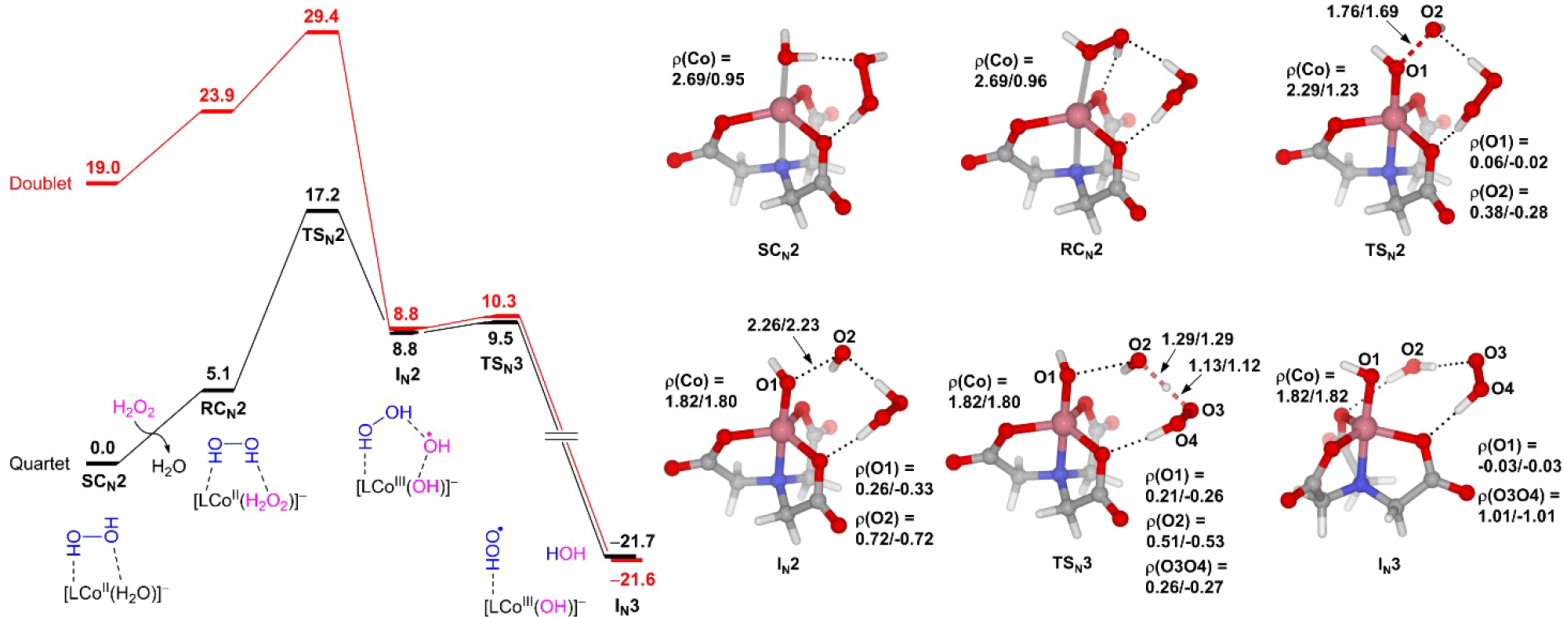
Free energy profile of the second-sphere H_2_O_2_-assisted Fenton-like reaction mediated by the NTA complex of Co­(II). Selected bond distances and spin densities (ρ) are given for quartet/doublet states. The energy unit is kcal/mol, and the bond distance unit is Å.

To assess the ability of H_2_O_2_ to compete with H_2_O for second-sphere coordination sites, the hydrogen bond strength between H_2_O/H_2_O_2_ and acetate was estimated, and the results are summarized in Figure S6. The calculations reveal that the hydrogen bond enthalpy of CH_3_CO_2_^–^···H_2_O_2_ is significantly greater than that of CH_3_CO_2_^–^···H_2_O (7.0 vs 4.4 kcal/mol). After considering the entropic effect and the concentration effect (1 M for H_2_O_2_ and 55.5 M for H_2_O), the hydrogen bond free energy of the two complexes becomes comparable (2.1 vs 2.4 kcal/mol). The results support the idea that H_2_O_2_ can compete with H_2_O for binding to the carboxylate groups of NTA– and EDTA–Co­(II) complexes.

### 3.2 Activation of H_2_O_2_ by EDTA–Co­(II) Complex

Figure 4 presents the results of the conventional Fenton-like reaction mediated by the EDTA complex of Co­(II). The EDTA ligand complexes with Co­(II) to form a six-coordinate octahedral complex [(EDTA)­Co^II^]^2–^. This complex has no exchangeable water ligands, and H_2_O_2_ must replace one of the carboxylate groups to coordinate with Co­(II) in order to be activated. To simulate this process, we start with the complex [(EDTA)­Co^II^]^2–^·H_2_O_2_ (**SC**_**E**_ **1**), in which H_2_O_2_ is attached to the carboxylate groups of EDTA via hydrogen bonds. Calculations show that H_2_O_2_ replaces the carboxylate group via an interchange mechanism to form the reactant complex [(EDTA)­Co^II^(H_2_O_2_)]^2–^ (**RC**_**E**_ **1**) without carboxylate dissociative or H_2_O_2_ associated intermediates (Figure S7). The following O–O bond cleavage was found to involve a spin transition from the HS quartet state to the LS doublet state after the transition state **TS**_**E**_ **2**, leading to the formation of the LS doublet intermediate ^2^[(EDTA)­Co^III^(OH)]^2–^·^•^OH (^**2**^ **I**_**E**_ **1**). In contrast to the NTA system where ^**2**^ **I**_**N**_ **1** and ^**4**^ **I**_**N**_ **1** possess similar electronic structures and energies (Figure 1), ^**2**^ **I**_**E**_ **1** and ^**4**^ **I**_**E**_ **1** are characterized by singlet state and triplet state [(EDTA)­Co^III^(OH)]^2–^ moieties [ρ­(Co) = 0.02 vs 1.76], respectively, and thus have rather different energies (Figure 4). The different electronic structures of ^**2**^ **I**_**E**_ and ^**4**^ **I**_**E**_ are a consequence of the d-orbital splitting of the octahedral Co­(II) complex (Figure 2).

**Figure 4:**
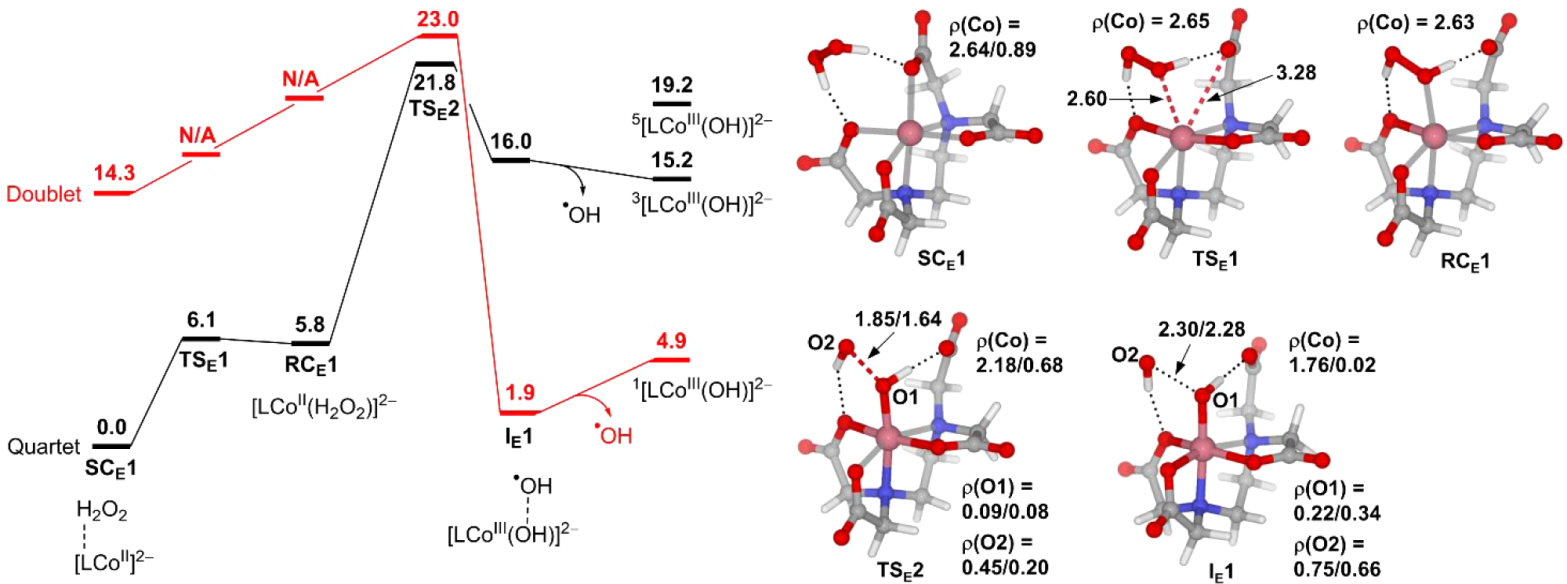
Free energy profile of the conventional Fenton-like reaction mediated by the EDTA complex of Co­(II). Selected bond distances and spin densities (ρ) are given for quartet/doublet states. The energy unit is kcal/mol, and the bond distance unit is Å.

Although the reaction energy of the conventional Fenton-like reaction mediated by the EDTA–Co­(II) complex is somewhat lower compared with the Co­(II)/H_2_O_2_/NTA system, the reaction is still endergonic and should not be the major H_2_O_2_ activation pathway. Therefore, we again consider the effect of second-sphere H_2_O_2_ on the reaction, and the calculated results are summarized in Figure 5. The results show that the presence of H_2_O_2_ in the second coordination sphere decreases the activation energy of O–O bond cleavage from 21.8 to 19.1 kcal/mol, and the subsequent HAT reaction between the H_2_O_2_ and the nascent ^•^OH provides the thermodynamic driving force, similar to the results of Co­(II)/H_2_O_2_/NAT system. As mentioned in the introduction, while ^•^OH was detected in the Co­(II)/H_2_O_2_/EDTA reaction, only a small amount of consumed H_2_O_2_ was converted to ^•^OH (10–15%). So, in addition to the conventional Fenton-like reaction, there exists an unknown major H_2_O_2_ activation pathway.^26^ According to the present calculations, we reasonably propose that this major activation pathway is the generation of ^•^OOH via the second-sphere H_2_O_2_-assisted Fenton-like reaction.

**Figure 5:**
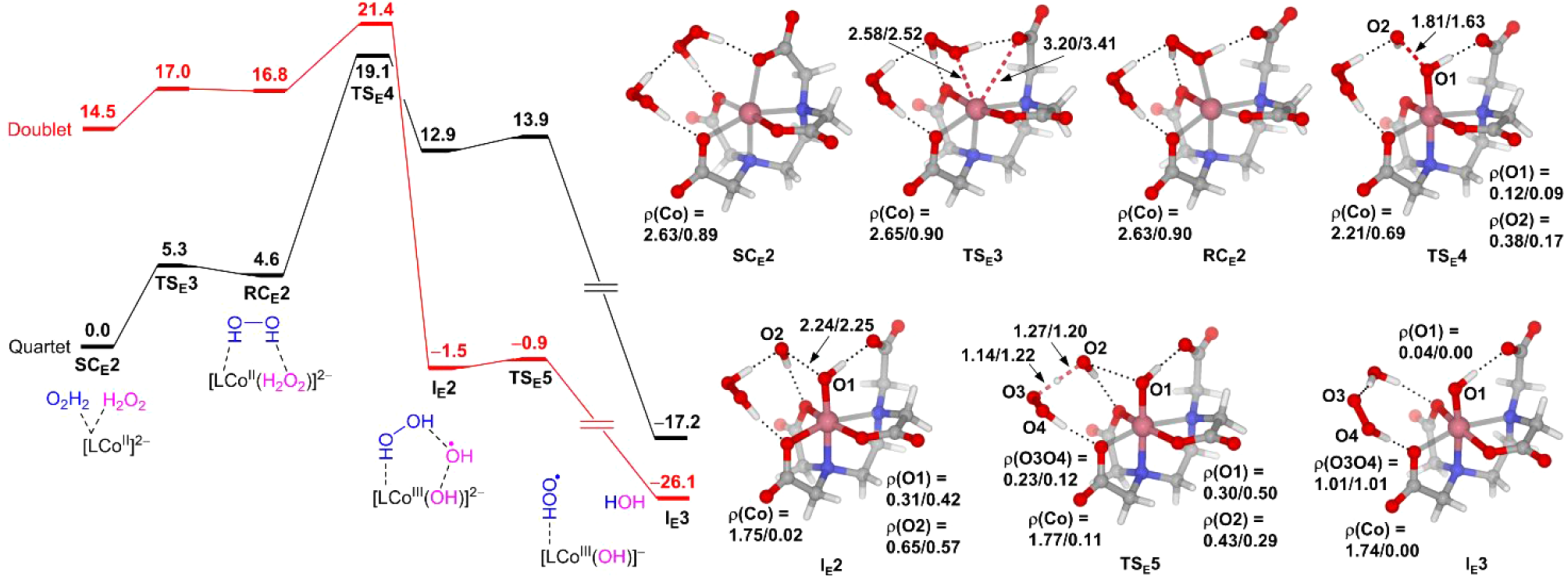
Free energy profile of the second-sphere H_2_O_2_-assisted Fenton-like reaction mediated by the EDTA complex of Co­(II). Selected bond distances and spin densities (ρ) are given for quartet/doublet states. The energy unit is kcal/mol, and the bond distance unit is Å.

In recent years, the effects of the second coordination sphere on reaction reactivity and selectivity have attracted much attention.^62–65^ Our second-sphere H_2_O_2_-assisted Fenton-like pathway can be regarded as a variant of the second coordination sphere effect, in which the functional group in the second coordination sphere is replaced by the reagent H_2_O_2_. The enhanced reactivity upon complexation is usually attributed to the regulation of the redox potential of metal ions by the ligand. However, the above calculations show that the complexation of NTA and EDTA cannot lower the redox potential of Co­(II) to a level sufficient to allow the conventional Fenton-like reaction to proceed spontaneously. On the other hand, the carboxylate groups of NTA and EDTA act as proton acceptors to attract H_2_O_2_ to the second coordination sphere through hydrogen bonding. The HAT from H_2_O_2_ in the second coordination sphere to ^•^OH produced by the coordinated H_2_O_2_, which is a highly exergonic reaction, provides an additional thermodynamic driving force for H_2_O_2_ decomposition. In other words, the decrease in redox potential upon complexation and the second-sphere H_2_O_2_ effect cooperatively endow these Co­(II) complexes with Fenton-like reactivity. The synergistic effect of these two factors may also be exploited to activate the Fenton-like reactivity of other redox couples with redox activity lower than that of Co­(II)/Co­(III). To verify this possibility, we performed preliminary calculations to estimate the reaction free energies of conventional Fenton-like (Reaction 4) and second-sphere H_2_O_2_-assisted Fenton-like reactions (Reaction 5) mediated by the NTA complexes of Ni­(II) and Cu­(II). As

4 [(NTA)MII(H2O)]−+H2O2→[(NTA)MIII(OH)]−+H2O+OH•

5 [(NTA)MII(H2O)]−+2H2O2→[(NTA)MIII(OH)]−+2H2O+OOH•

expected, Reaction 4 of the Ni­(II) and Cu­(II) complexes is thermodynamically unfavorable, with endergonicities of 12.4 and 21.4 kcal/mol, respectively. However, Reaction 5 of the Ni­(II) and Cu­(II) complexes becomes exergonic by −18.6 and −9.5 kcal/mol, respectively. It is important to emphasize that although the reaction of ^•^OH with H_2_O_2_ to form H_2_O and ^•^OOH is predicted to be a highly exergonic reaction based on bond energy considerations, the increased or dominant formation of ^•^OOH/O_2_^•–^ is not a universal phenomenon in Fenton and Fenton-like systems. The key ingredient is likely the accumulation of H_2_O_2_ around the active center. Based on these considerations, we propose that selecting transition metal ions with high redox potentials to avoid conventional Fenton-like reactions, in combination with the use of ligands containing available proton-accepting sites to attract H_2_O_2_ and promote HAT from second-sphere H_2_O_2_ to coordinated H_2_O_2_ may be an effective strategy to achieve selective production of ^•^OOH/O_2_^•–^.

According to the Wigner spin conservation rule, reactions occurring on a single spin potential surface are spin-allowed, while reactions involving spin state changes are spin-forbidden and should be slow processes. Therefore, one may question whether the spin state change proposed in the mechanisms can occur. Thirty years ago, Shaik proposed an important concept of *two-state reactivity* (TSR), which describes the phenomenon that the minimum-energy pathway of a reaction is determined by two states of different spin multiplicities.^66,67^ The TSR mechanism has been successfully used to analyze and understand the hydrogen abstraction reactivity of iron­(IV)-oxo complexes.^68,69^ Very recently, the correlation between spin state energy gaps and C–H bond activation rates has been established,^70^ and the spin state change during the heterogeneous Fenton-like reaction has been monitored by magnetic measurements.^71^ These previous studies support the idea that spin transitions can occur in Co­(II)-mediated Fenton-like reactions.

### 3.3 Activation of H_2_O_2_ by GSH–Co­(II) Complex

The coordination modes of the cobalt ion with GSH are relatively complicated due to the presence of diverse functional groups in GSH (Figure 6). The binding of Co­(II) and Co­(III) to GSH has been investigated by X-ray absorption spectroscopy (XAS) in combination with DFT calculations^72^ and Car–Parrinello molecular dynamics (CPMD) simulations.^73^ The pre-edge features of the X-ray absorption near-edge structure (XANES) spectra of GSH–Co­(II) and GSH–Co­(III) complexes are typical for an octahedral environment. The fitting of extended X-ray absorption fine structure (EXAFS) spectra indicated that Co­(II) and Co­(III) were surrounded by one S and five N/O atoms.^72^ DFT calculations of the GSH–Co­(III) complex suggested that the six-coordinated structure [(GSH)­Co^III^(OH)]^−^, in which GSH chelates with Co­(III) using N1, O1, N2, O4, and S atoms (cf. Figure 6 for atom labels) with one OH^–^ completing the coordination sphere, showed the best agreement with EXAFS data.^72^ Another DFT and CPMD study of the GSH–Co­(II) complex revealed a four-coordinate structure. However, this study did not consider the coordination of H_2_O.^73^ Here, the binding modes of Co­(II) with GSH were reinvestigated. Three optimized structures of the six-coordinate [(GSH)­Co^II^(H_2_O)]^−^ complex were found and depicted in Figure 6. Among these conformations, the most stable one is N1O1N2O3S, in which GSH forms a five-coordinate structure with Co­(II) using the amine and carboxylate groups of the glutamate moiety and the amine, carbonyl, and thiolate groups of the cysteine moiety, with one water molecule occupying the vacant site. This lowest-energy structure is consistent with the results of XANES and EXAFS, that is, Co­(II) is surrounded by one S and five N/O atoms, and is used as a starting complex for the following study of the conventional Fenton-like reaction.

**Figure 6:**
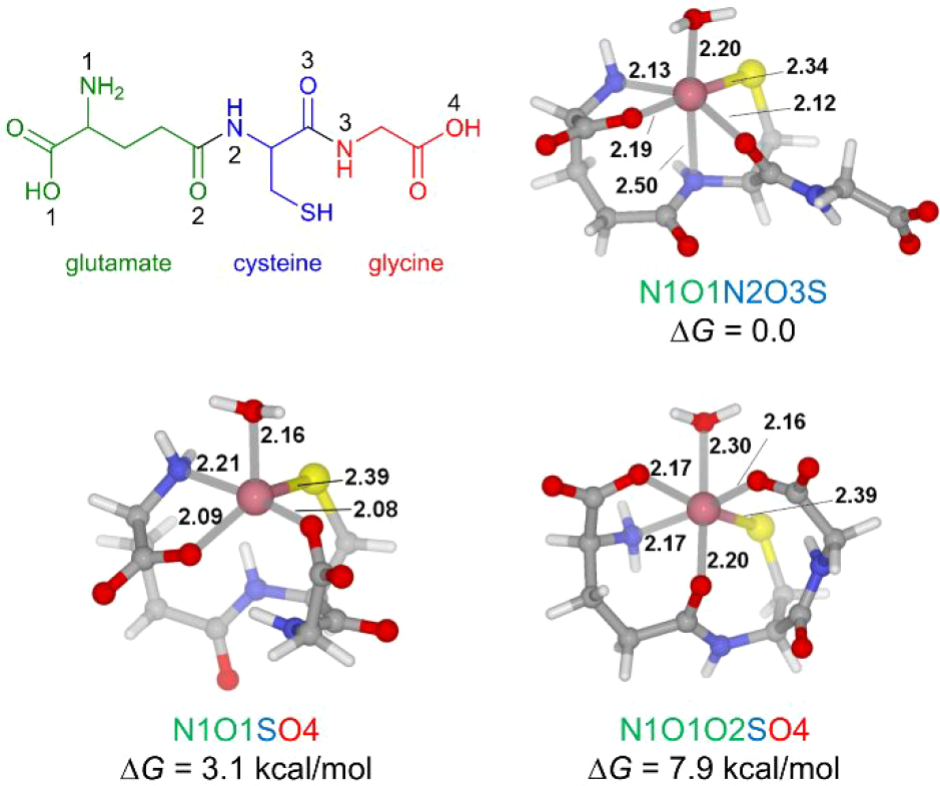
Optimized structures of [(GSH)­Co^II^(H_2_O)]^−^. The energy unit is kcal/mol, and the bond distance unit is Å.

Figure 7 presents the calculated free energy profile of a conventional Fenton-like reaction mediated by the N1O1N2O3S complex (i.e., **SC**_**G**_). The results show that GSH complexation significantly promotes the Co­(II)-mediated Fenton-like reaction both in terms of kinetics and thermodynamics. Given that the GSH–Co­(II) complex possesses a similar coordination environment to the NTA– and EDTA–Co­(II) complexes, except for the thiolate group, it appears that the thiolate group plays a major role in promoting the Fenton-like reaction. To confirm this point, we calculated the Fenton-like reaction of the modified GSH complex, in which the thiolate group was replaced by the hydroxyl group to mimic the coordination of H_2_O. The results show that without thiolate coordination, the Fenton-like reaction becomes highly unfavorable indeed (cf. Figure S8a and b). Analogous calculations, in which each coordinating functional group of GSH was replaced by a hydroxyl group, were also performed to check their individual effect on the reaction. The order of ability to facilitate the reaction was found to be thiolate > amine > amide nitrogen > carboxylate > carbonyl (Figure S8). In fact, the carbonyl group exhibits a negative effect on the Fenton-like reaction relative to the hydroxyl group (Figure S8e).

**Figure 7:**
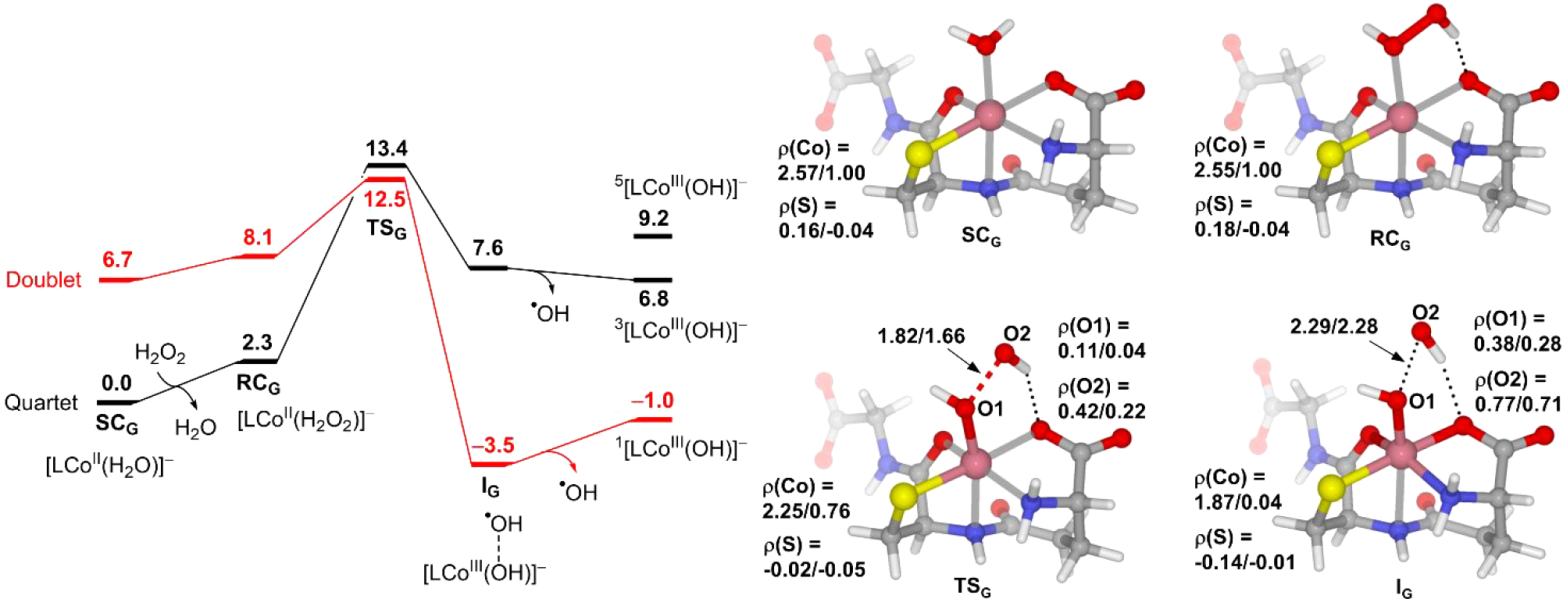
Free energy profile of the conventional Fenton-like reaction mediated by the GSH complex of Co­(II). Selected bond distances and spin densities (ρ) are given for quartet/doublet states. The energy unit is kcal/mol, and the bond distance unit is Å.

The next question is how does the thiolate group promote the Fenton-like reaction? One of the effects of the thiolate group is to provide a larger stabilization to the transition state (**TS**_**G**_) and the Co­(III) complex [LCo^III^(OH)]^−^·^•^OH (**I**_**G**_) than to the Co­(II) complex [LCo^II^(H_2_O)]^−^ (**SC**_**G**_), resulting in a decrease in activation and reaction energies. Comparing the results for the GSH complex (right in Figure 8) and the modified GSH complex, in which −S^–^ is replaced by −OH (left in Figure 8) indicates that this effect lowers the activation and reaction energies by 9.5 and 11.9 kcal/mol in the HS quartet state and by 7.3 and 9.1 kcal/mol in the LS doublet state, respectively. The free energy profile in the middle of Figure 8 was constructed by adding these decreasing values to the ^**2/4**^ **TS**_**G**_′ and ^**2/4**^ **I**_**G**_′ of the modified GSH system while fixing the HS–LS energy gap of the starting complex [LCo^II^(H_2_O)]^−^ (**SC**) at 13.7 kcal/mol. It can be seen that the stabilization effect greatly facilitates the reaction kinetics, but this effect alone is not sufficient to make the reaction thermodynamically favorable. The second role of the thiolate group is to destabilize the HS state relative to that of the LS state. This effect originates from the strong interaction between the electron-rich thiolate group and the d orbitals of cobalt ions. In the simple ligand-field description of octahedral complexes, the consequence of this thiolate-metal ion interaction is to raise the antibonding e_g_ sets, which are occupied by more electrons in HS states than in LS states, resulting in a relative destabilization of the former with respect to the latter. This effect raises the HS free energy surface or, from another perspective, lowers the LS free energy surface by 7 kcal/mol, rendering the reaction exergonic (cf. middle and right free energy profiles in Figure 8). In fact, the destabilization of the HS state relative to the LS state by thiolate ligands has been recognized to play a critical role in altering the spin state, thereby regulating the redox potential and enzymatic activity of cytochrome P450.^74–77^

**Figure 8:**
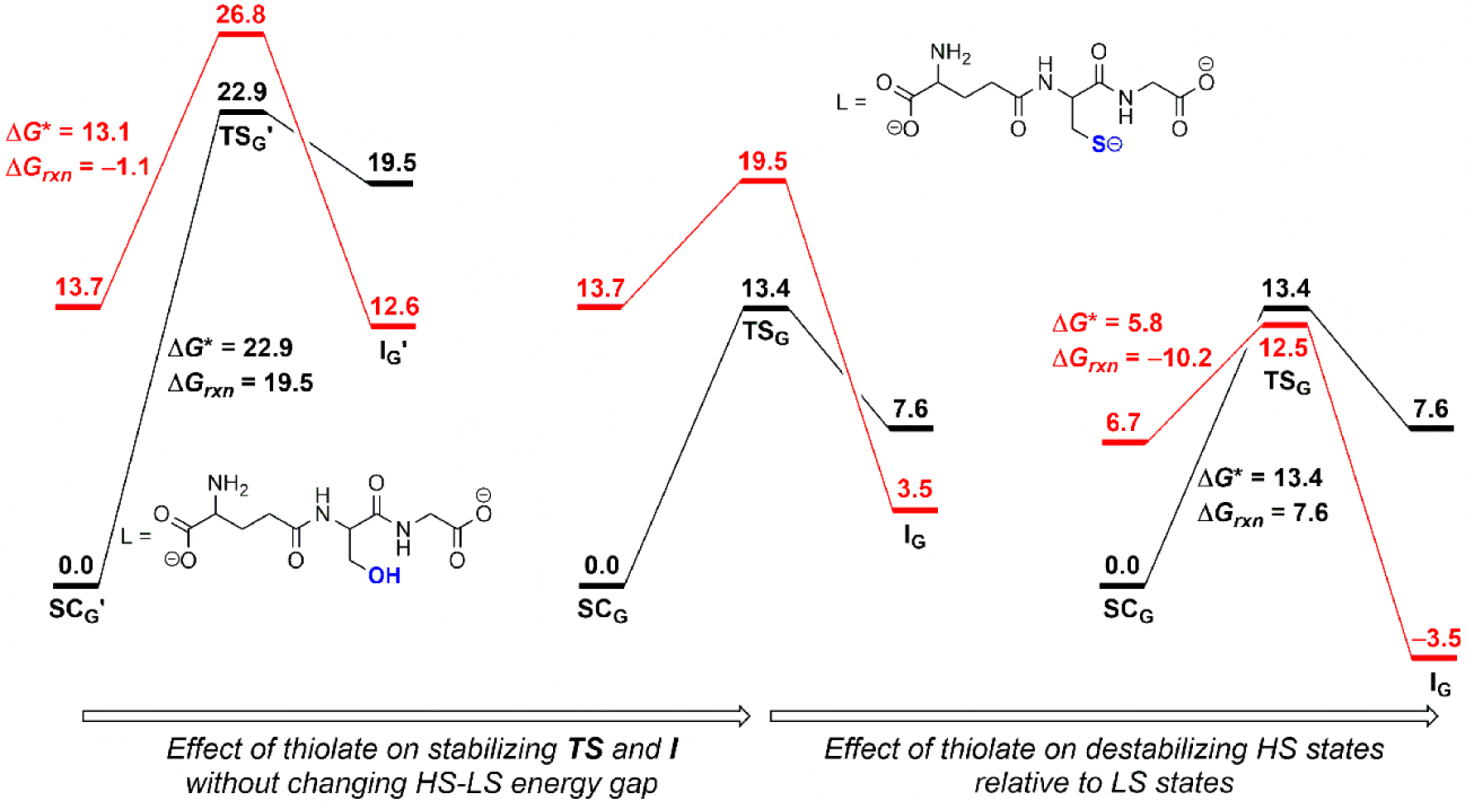
Effects of the thiolate group on the Fenton-like reaction. Black and red indicate quartet and doublet states, respectively.

GSH is an important biomolecule. It can act directly as an antioxidant to protect cells from free radical damage or act as a cofactor for antioxidant and detoxification enzymes. Nevertheless, our calculations show that the GSH–Co­(II) complex acts as a pro-oxidant, reacting with H_2_O_2_ to produce ^•^OH through a conventional Fenton-like reaction. Further analyses reveal that the thiolate group of GSH plays a critical role in promoting this reaction. These findings have two important implications. First, complexation with biomolecules containing thiolate groups can induce the Fenton toxicity of Co­(II) ions. Second, introducing thiolate groups into ligands can serve as an effective strategy for achieving selective ^•^OH generation in cobalt-based Fenton-like systems.

Finally, we point out that the three ligands employed in this study endow the complexes with different degrees of fluxionality. Fluxionality of polyhedral can provide a variety of rearrangement options,^78^ and low-energy nonideal polyhedral structures may also participate in the reaction. However, since the calculations of the most stable structures have already provided important mechanistic insights and explained experimental observations, we have not further considered Fenton-like reactions mediated by other low-energy polyhedral structures.

### 3.4 Reaction of NTA–Co­(III) Complex with DMPO

As mentioned in the introduction, EPR spin trapping experiments using the DMPO reagent reported the formation of ^•^OOH and ^•^OH in the Co­(II)/H_2_O_2_/NTA system. However, according to the present calculations, the formation of free ^•^OH from the conventional Fenton-like reaction between [(NTA)­Co^II^(H_2_O)]^−^ and H_2_O_2_ was found to be thermodynamically unfavorable with an endergonicity of about 9 kcal/mol (Figure 1). Furthermore, the products of the second-sphere H_2_O_2_-assisted Fenton-like pathway are ^•^OOH and [(NTA)­Co^III^(OH)]^−^ without ^•^OH (Figure 3).

In our previous study on the reaction between [Co^II^(H_2_O)_6_]^2+^ and H_2_O_2_, we discovered that the generated Co­(III) species displayed moderate to strong oxidation reactivity.^25^ This finding inspired us to investigate the possibility of DMPO oxidation by [(NTA)­Co^III^(OH)]^−^. Figure 9 presents the computational results of the reaction between [(NTA)­Co^III^(OH)]^−^ and DMPO. We found that [(NTA)­Co^III^(OH)]^−^ could transfer the OH group to DMPO. Spin population analysis indicates that during this process Co­(III) is reduced to Co­(II), producing a product complex consisting of the HS quartet [(NTA)­Co^II^]^−^ antiferromagnetically coupled with DMPO–^•^OH. This reaction is spontaneous and rapid, with an activation energy of only 15.7 kcal/mol.

**Figure 9:**
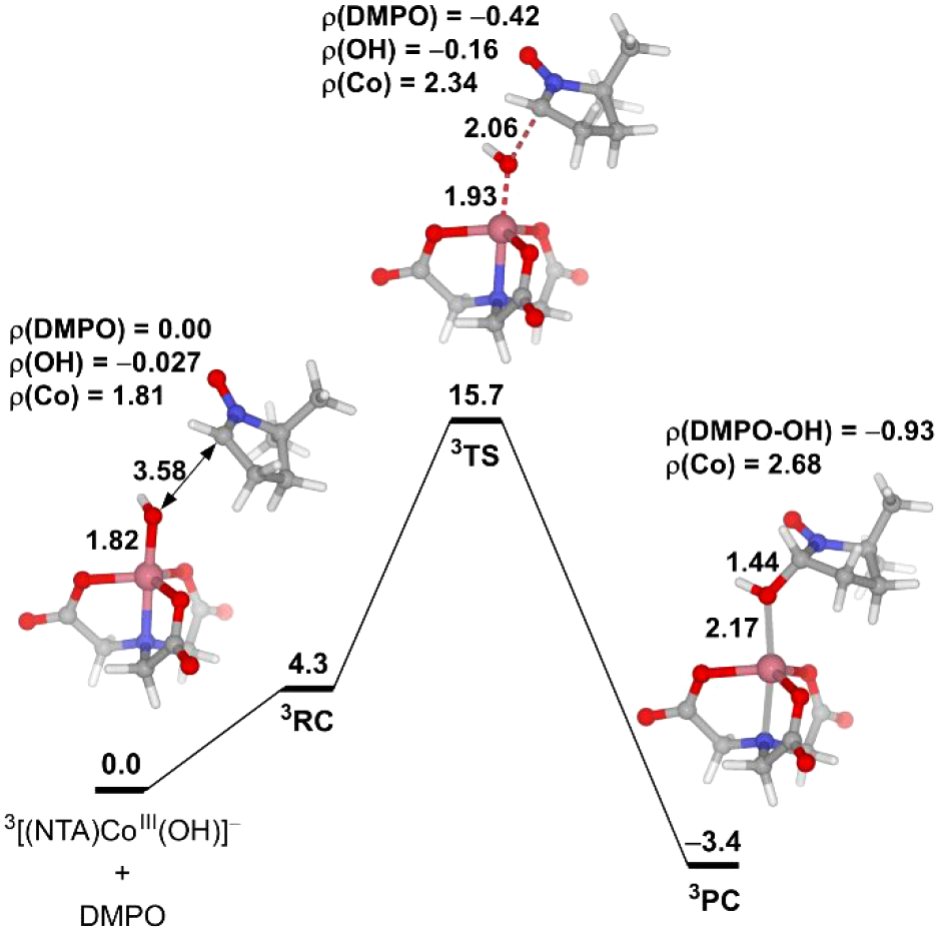
Free energy profile of the reaction between [(NTA)­Co^III^(OH)]^−^ and DMPO. The energy unit is kcal/mol, and the bond distance unit is Å.

We also considered the possibility of generating the DMPO–^•^OH adduct and [(NTA)­Co^III^(OH)]^−^ by the reaction of [(NTA)­Co^II^(H_2_O_2_)]^−^ with DMPO, like the second-sphere H_2_O_2_-assisted Fenton-like reaction shown in Figure 3, but now the H_2_O_2_ in the second coordination sphere is replaced by DMPO. This reaction was calculated to be highly exergonic with an activation energy of 22.5 kcal/mol (Figure S9). The activation energy of this reaction is much higher than that of the reaction between [(NTA)­Co^III^(OH)]^−^ and DMPO, and therefore, it cannot compete in kinetics.

Based on the above results, we conclude that the EPR signal of the DMPO–^•^OH adduct detected in the Co­(II)/H_2_O_2_/NTA reaction does not indicate the formation of free ^•^OH; in fact, this signal mainly originates from the reaction of [(NTA)­Co^III^(OH)]^−^ with DMPO. EPR spin trapping with the DMPO reagent is widely used to detect the generation of free ^•^OH in diverse fields, such as biology, materials science, and environmental science. The present calculations suggest that this technique should be used with caution when studying reactions containing or generating Co­(III) species.

## 4 Conclusions

In summary, the present study reveals that aminopolycarboxylic NTA and EDTA ligands cannot reduce the redox potential of Co­(II) to a level sufficient to promote ^•^OH formation through conventional Fenton-like reactions. The reductive HO–OH bond cleavage in these complexes requires the aid of hydrogen atom transfer from H_2_O_2_ attached to the carboxylate group through the hydrogen bond. This second-sphere H_2_O_2_-assisted Fenton-like reaction could be used to selectively produce ^•^OOH/O_2_^•–^. In contrast, the complexation of glutathione directly promotes the conventional Co­(II)-mediated Fenton-like reaction, generating ^•^OH as the major ROS. The thiolate group plays a critical role in promoting this reaction, suggesting that introducing a thiolate group into the ligand may be an effective strategy for achieving the selective generation of ^•^OH. Another implication is that the Fenton toxicity of Co­(II) may be enhanced upon complexation with biomolecules, especially those containing thiolate groups. Finally, our calculations demonstrate that [(NTA)­Co^III^(OH)]^−^ can react with DMPO to form the DMPO–^•^OH adduct, which raises a caveat for using the EPR spin trapping technique to detect the formation of free ^•^OH in the presence of Co­(III) ions.

## Supplementary Material





## Notes

Potential energy scan of side-on H_2_O_2_ in the NTA complex; potential energy scan of water ligand dissociation; results of the hydrogen bond between acetate and H_2_O/H_2_O_2_; potential energy scan of H_2_O_2_ coordination in [(EDTA)­Co^II^]^2–^·H_2_O_2_; free energy profiles of modified GSH–Co­(II) complexes; free energy profile of the reaction between [(NTA)­Co^II^(H_2_O_2_)]^−^ and DMPO (PDF)
DFT optimized coordinates (XYZ)
