# Supplementary material for: Cobalt(II)-Mediated Fenton-like Reactions: Effects of Second-Sphere H2O2 and Thiolate Coordination
Source: Inorg Chem. 2025 Dec 16;65(1):557–67. doi: 10.1021/acs.inorgchem.5c04687 (PMC12801387; doi:10.1021/acs.inorgchem.5c04687)
Supplement: Supplementary file 1 [file ic5c04687_si_001.pdf]

Supporting Information

Cobalt(II)-Mediated Fenton-like Reactions: Effects of Second-Sphere H_2O_2 and Thiolate Coordination

Hsing-Yin Chen* and Yu-Fen Lin

Department of Medicinal and Applied Chemistry, Kaohsiung Medical University, Kaohsiung 80708, Taiwan

E-mail: hychen@kmu.edu.tw

Table of Contents

- Figure S1. Potential energy surface scan of H_2O_2 approaching Co(II) center of NTA complex via a side-on manner.
- Figure S2. Potential energy surface scan of water ligand dissociation of $[(NTA)Co^{II}(H_2O)]^-$.
- Figure S3. Potential energy surface scan of water ligand dissociation of $[(NTA)Co^{II}(H_2O)]^-\cdot H_2O_2$.
- Figure S4. Potential energy surface scan of water ligand dissociation of $[(NTA)Co^{II}(H_2O)]^-\cdot 2H_2O_2$.
- Figure S5. Potential energy surface scan of water ligand dissociation of $[(GSH)Co^{II}(H_2O)]^-$.
- Figure S6. Hydrogen bonding structures and energies of acetate with H_2O and H_2O_2.
- Figure S7. Potential energy surface scan of H_2O_2 entering the first coordination sphere from the second coordination sphere of $[(EDTA)Co^{II}]^{2-}\cdot H_2O_2$.
- Figure S8. Free energy profiles of conventional Fenton-like reactions mediated by modified GSH-Co(II) complexes.
- Figure S9. Free energy profile of reaction between $[(NTA)Co^{II}(H_2O_2)]^-$ and DMPO.

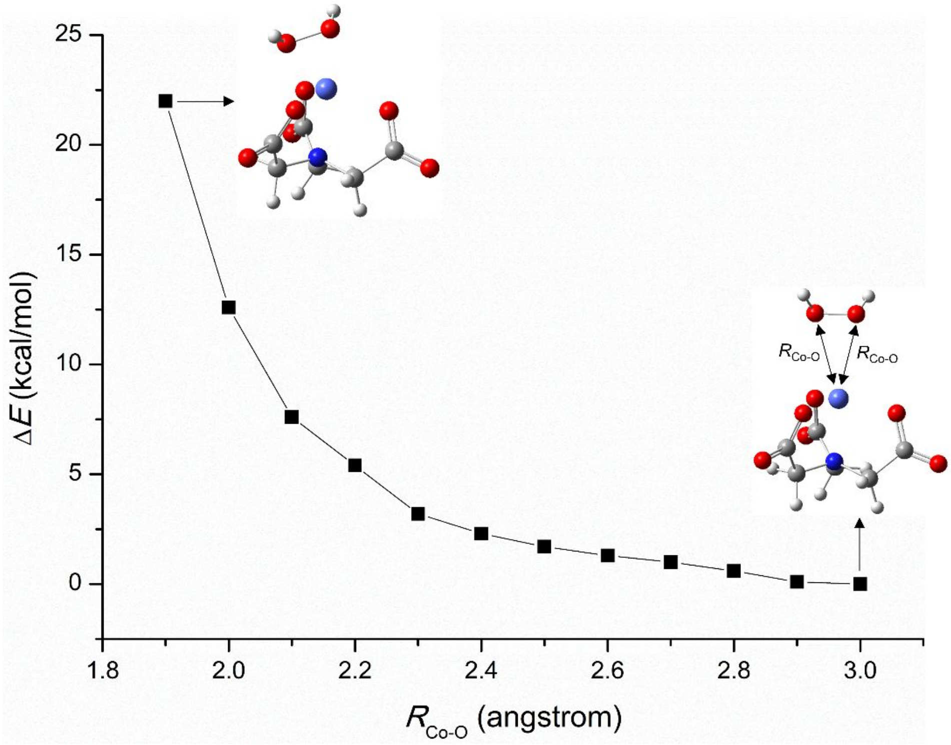

Figure S1. Potential energy surface scan of H_2O_2 approaching Co(II) center of NTA complex via a side-on manner.

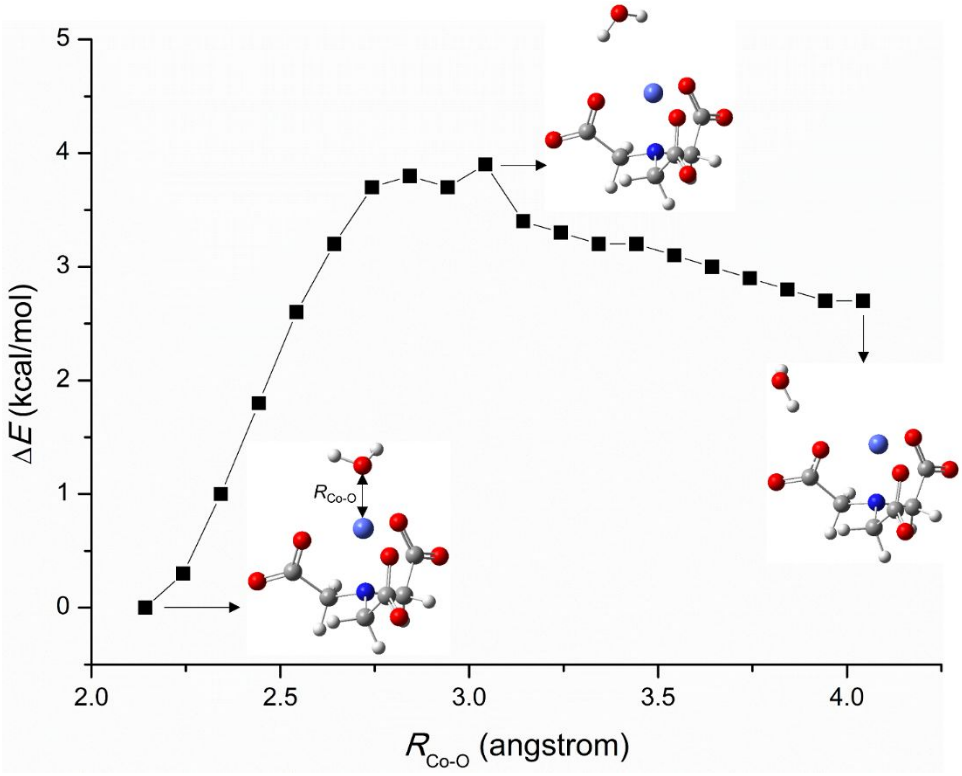

Figure S2. Potential energy surface scan of water ligand dissociation of $[(NTA)Co^{II}(H_2O)]^{-}$.

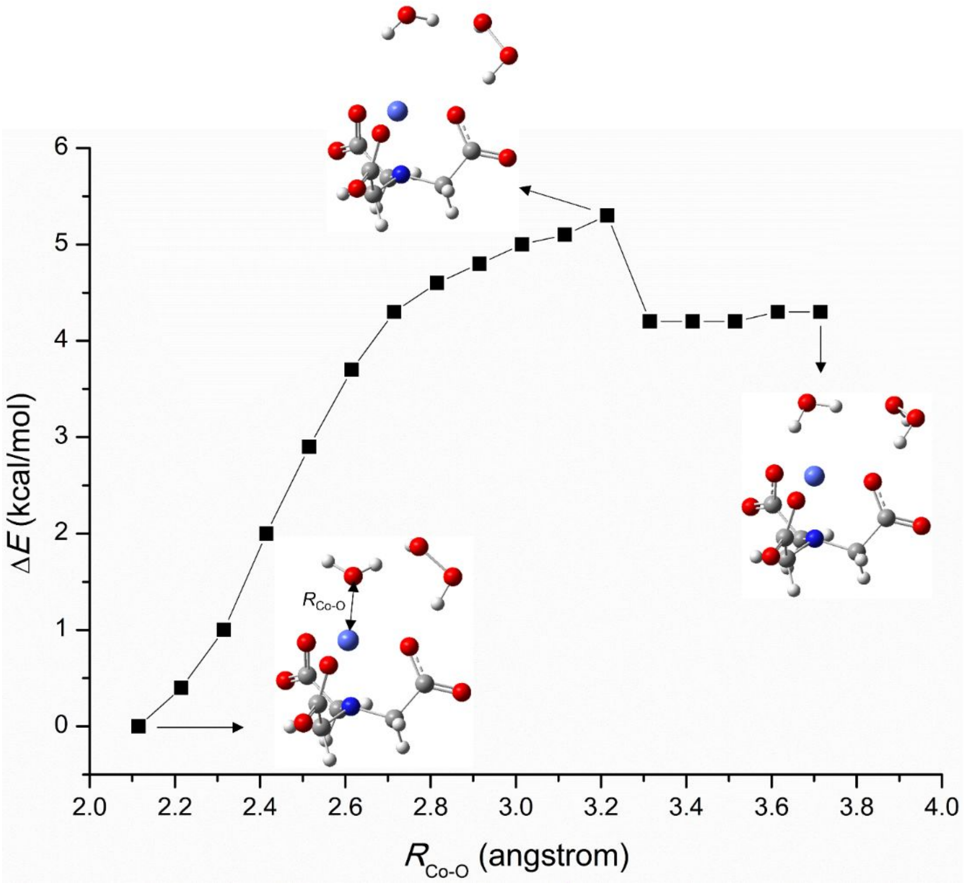

Figure S3. Potential energy surface scan of water ligand dissociation of $[(NTA)Co^{II}(H_2O)]^{-}\cdot H_2O_2$.

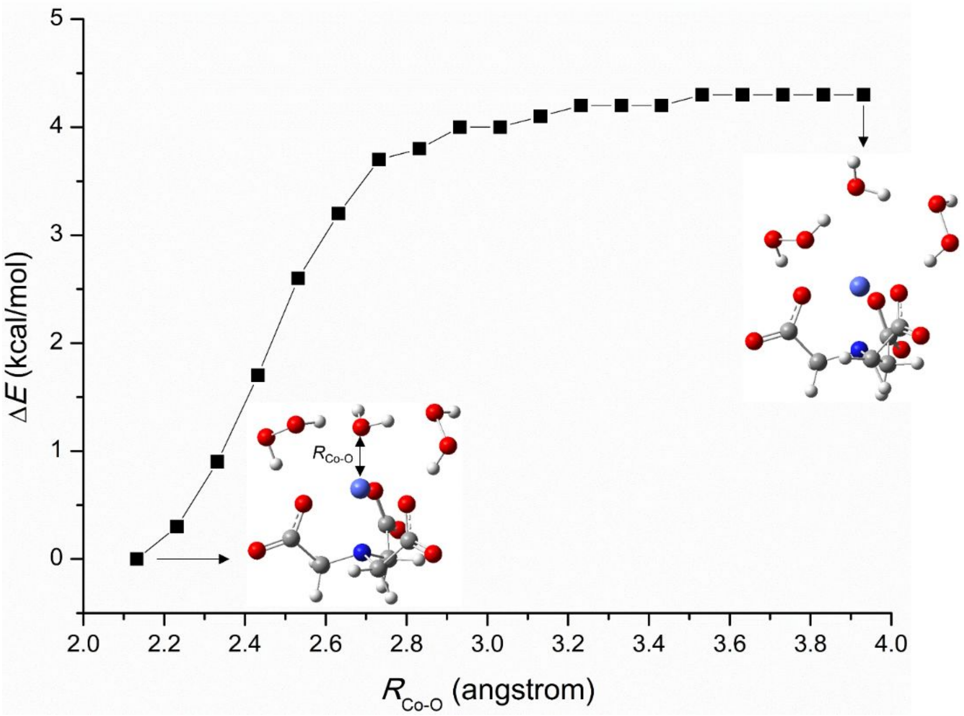

Figure S4. Potential energy surface scan of water ligand dissociation of $[(NTA)Co^{II}(H_2O)]^{-}\cdot 2H_2O_2$.

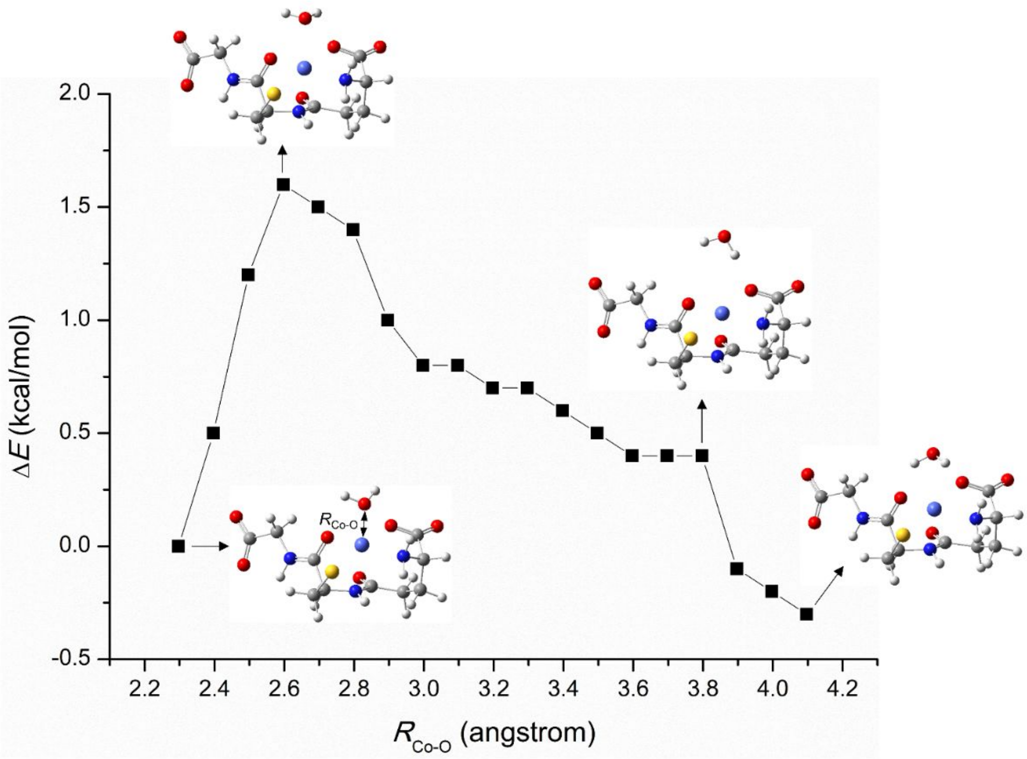

Figure S5. Potential energy surface scan of water ligand dissociation of $[(GSH)Co^{II}(H_2O)]^{-}$.

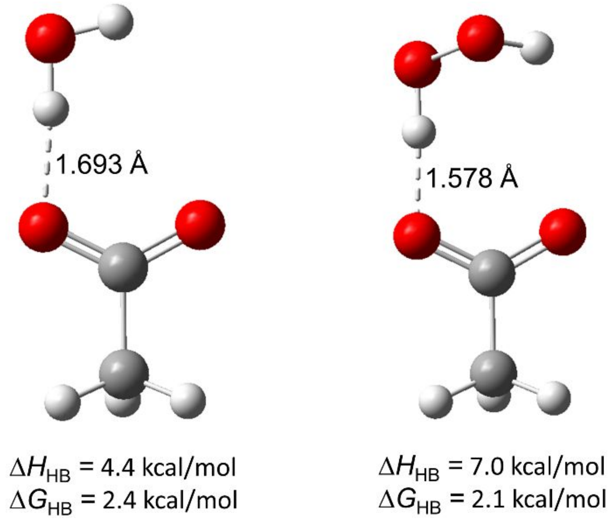

Figure S6. Hydrogen bonding structures and energies of acetate with H_2O and H_2O_2.

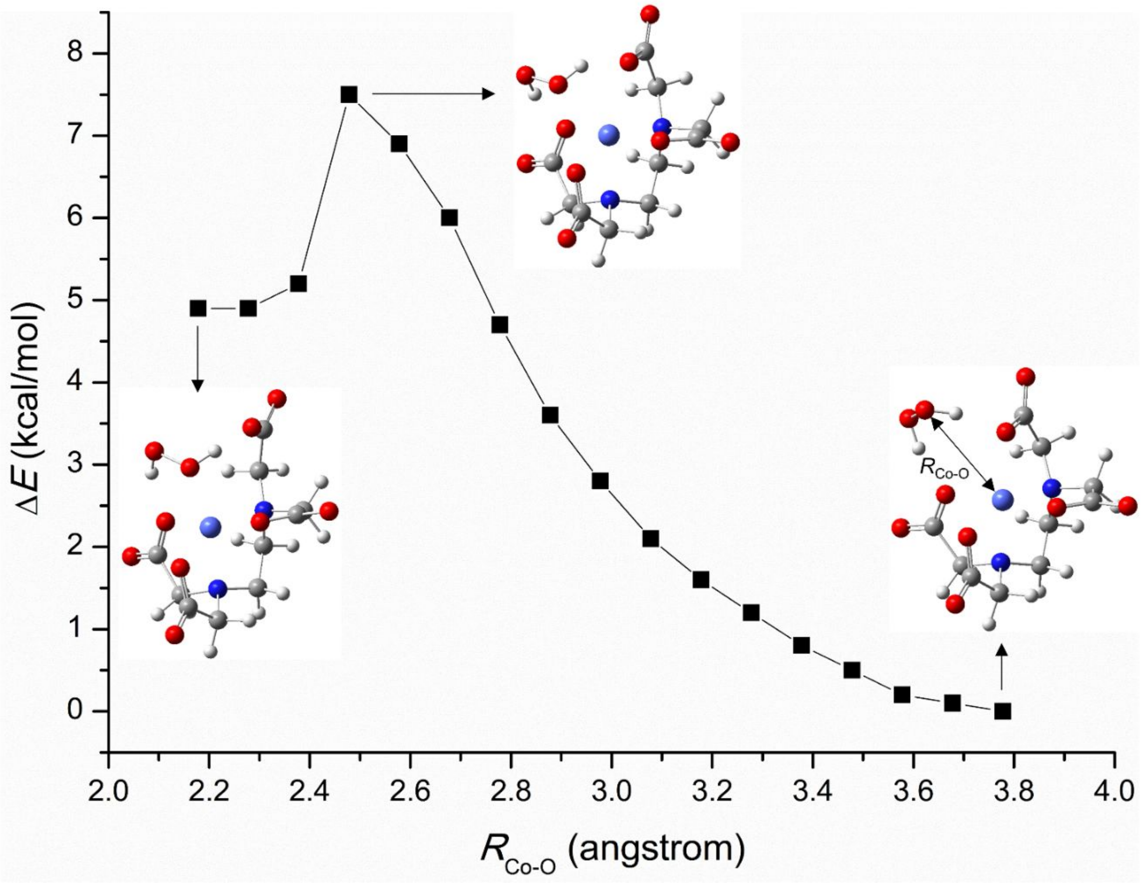

Figure S7. Potential energy surface scan of H_2O_2 entering the first coordination sphere from the second coordination sphere of $[(EDTA)Co^{II}]^{2-}\cdot H_2O_2$.

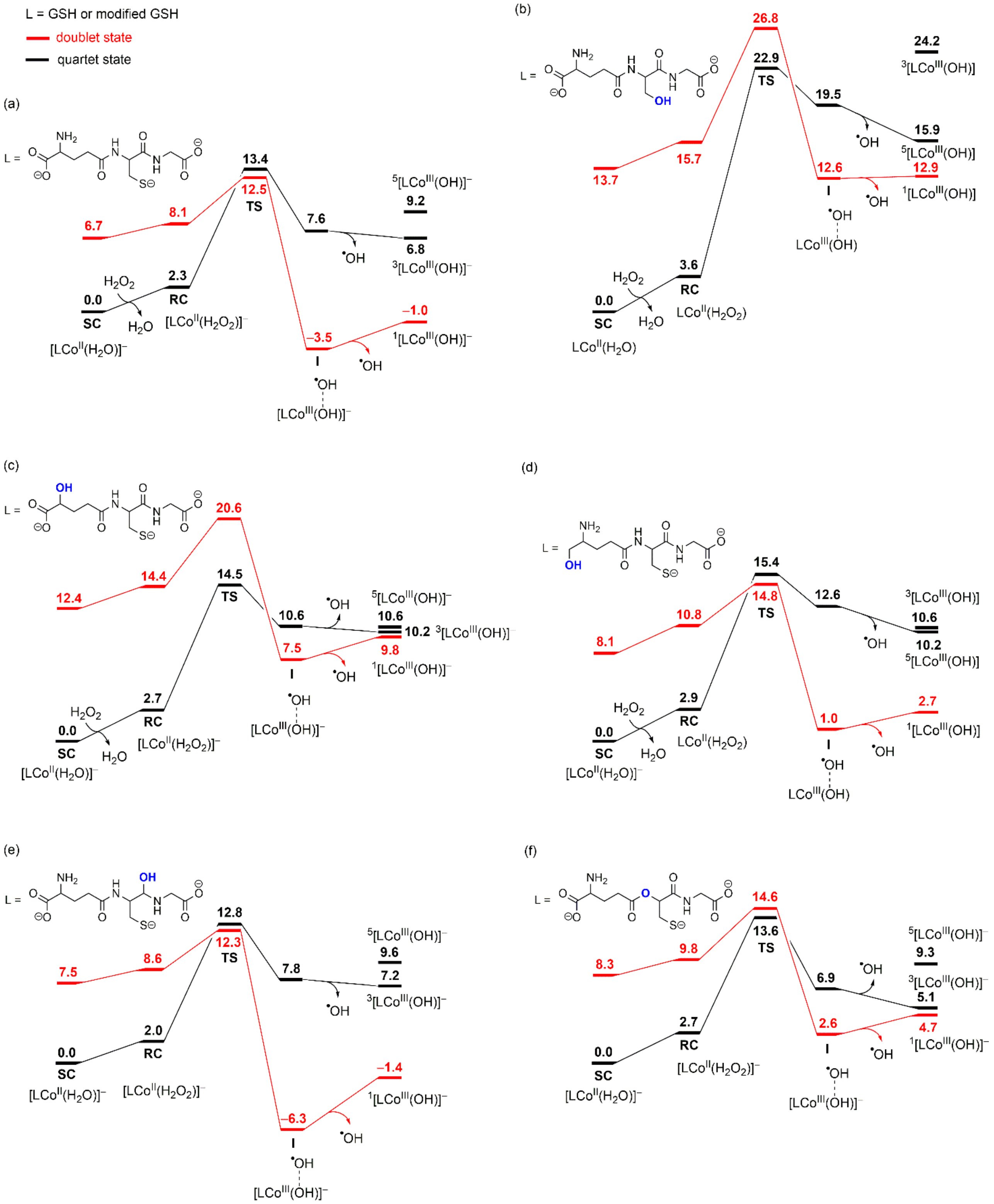

Figure S8. Free energy profiles of conventional Fenton-like reactions mediated by Co(II) complexes of (a) GSH (b) modified GSH with $-S^-$ replaced by –OH (c) modified GSH with $-NH_2$ replaced by –OH (d) modified GSH with $-CO_2^-$ replaced by $-CH_2OH$ (e) modified GSH with >C=O replaced by >CH–OH (f) modified GSH with –NH– replaced by –O–. Black and red indicate quartet and doublet states, respectively. Energy unit is kcal/mol.

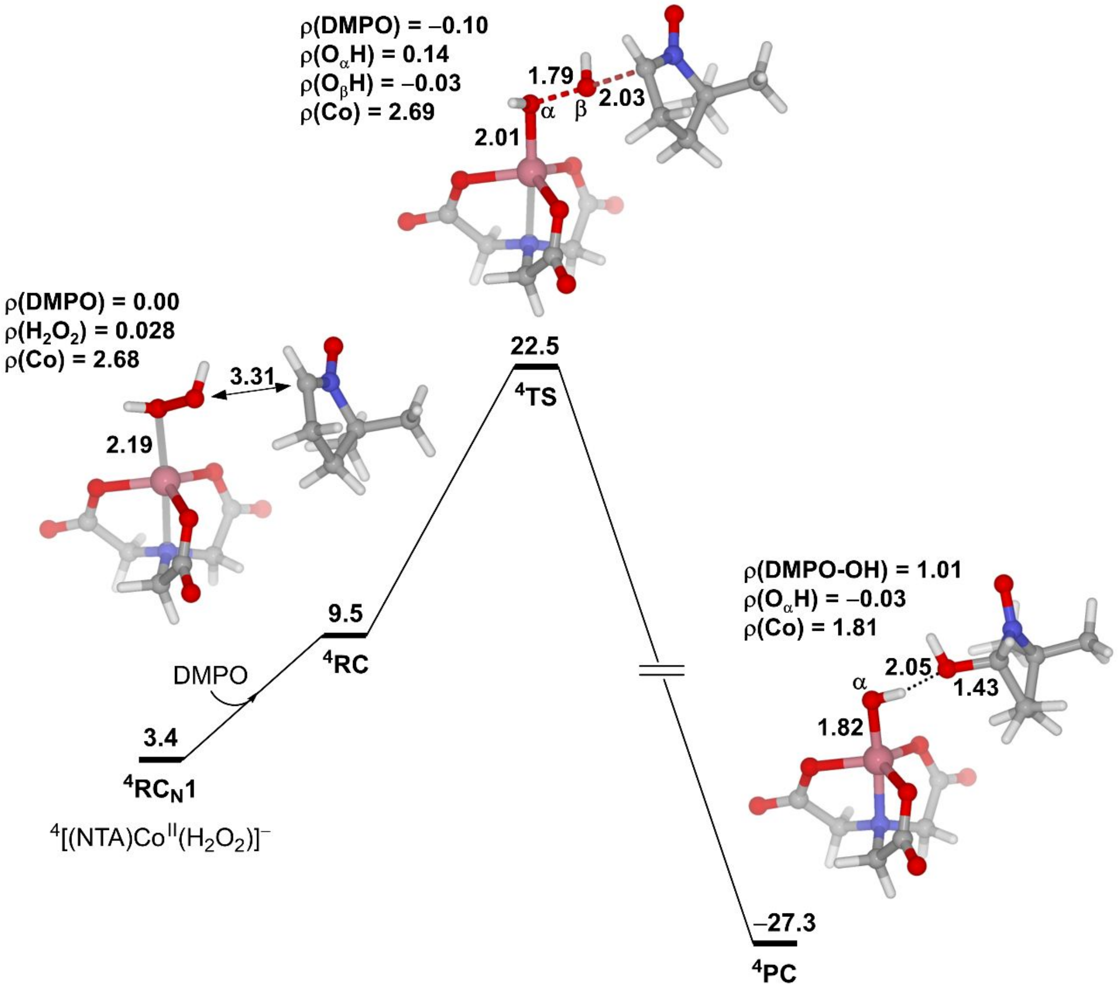

Figure S9. Free energy profile of reaction between $[(NTA)Co^{II}(H_2O_2)]^-$ and DMPO. Energy unit is kcal/mol and bond distance unit is Å.
